# Serine-Rich Repeat Adhesins Contribute to *Streptococcus gordonii*-Induced Maturation of Human Dendritic Cells

**DOI:** 10.3389/fmicb.2017.00523

**Published:** 2017-03-31

**Authors:** Eun Byeol Ko, Sun Kyung Kim, Ho Seong Seo, Cheol-Heui Yun, Seung Hyun Han

**Affiliations:** ^1^Department of Oral Microbiology and Immunology, DRI, and BK21 Plus Program, School of Dentistry, Seoul National UniversitySeoul, South Korea; ^2^Biotechnology Research Division, Korea Atomic Energy Research InstituteJeongeup, South Korea; ^3^Department of Agricultural Biotechnology and Research Institute for Agriculture and Life Sciences, Seoul National UniversitySeoul, South Korea

**Keywords:** *Streptococcus gordonii*, serine-rich repeat adhesins, dendritic cells, maturation, T cell activation

## Abstract

Dendritic cells (DCs) play a pivotal role in the induction of immunity by recognition, capture, process, and presentation of antigens from infectious microbes. *Streptococcus gordonii* is able to cause life-threatening systemic diseases such as infective endocarditis. Serine-rich repeat (SRR) glycoproteins of *S. gordonii* are sialic acid-binding adhesins mediating the bacterial adherence to the host and the development of infective endocarditis. Thus, the SRR adhesins are potentially involved in the bacterial adherence to DCs and the maturation and activation of DCs required for the induction of immunity to *S. gordonii*. Here, we investigated the phenotypic and functional changes of human monocyte-derived DCs treated with wild-type *S. gordonii* or the SRR adhesin-deficient mutant. The mutant poorly bound to DCs and only weakly increased the expression of CD83, CD86, MHC class II, and PD-L1 on DCs compared with the wild-type. In addition, the mutant induced lower levels of TNF-α, IL-6, and IL-12 than the wild-type in DCs. When DCs sensitized with the mutant were co-cultured with autologous T cells, they induced weaker proliferation and activation of T cells than DCs stimulated with the wild-type. Blockade of SRR adhesin with 3′-sialyllactose markedly reduced *S. gordonii* binding and internalization, causing attenuation of the bacterial immunostimulatory potency in DC maturation. Collectively, our results suggest that SRR adhesins of *S. gordonii* are important for maturation and activation of DCs.

## Introduction

*Streptococcus gordonii* is a Gram-positive facultative anaerobic bacterium belonging to the viridans group of oral streptococci (Loo et al., 2000). Although *S. gordonii* is a part of the normal flora in the oral cavity, it is able to cause various infectious diseases such as septic arthritis (Yombi et al., 2012) and life-threatening infective endocarditis with high mortality (Keane et al., 2010) through systemic spread following tooth extraction, brushing, or flossing (Forner et al., 2006). Upon entering the bloodstream, *S. gordonii* preferentially binds to human platelets, causing their aggregation, facilitating bacterial colonization in the endocardium and heart valves and resulting in endocarditis (Takahashi et al., 2006). Previous studies have shown that oral streptococci associated with endocarditis promote rapid differentiation of monocytes into mature dendritic cells (DCs) (Hahn et al., 2005) and *S. gordonii* induces the secretion of cytokines including TNF-α, IL-6, and IL-12 in DCs (Corinti et al., 1999), implying the importance of DCs in the disease development and immune responses.

Bacterial adherence is an important step for microbial pathogenesis (Moschioni et al., 2010). As one of the initial colonizers of dental biofilms, *S. gordonii* abundantly expresses diverse adhesins that mediate its binding to host tissues. *S. gordonii* utilizes serine-rich repeat (SRR) adhesins, antigen I/II family proteins, cell-surface fibrillar proteins, and amylase-binding proteins to bind to human platelets and monocytes (Takahashi et al., 2004; Urano-Tashiro et al., 2012). Among them, SRR adhesins play an important role in the development of infective endocarditis (Xiong et al., 2008; Jakubovics et al., 2009). *S. gordonii* adheres to sialic acids on platelets or erythrocytes through SRR adhesins in injured heart valves, exacerbating inflammatory responses by promoting deposition of bacterium-platelet-fibrin complexes and recruiting inflammatory immune cells in tissues (Yajima et al., 2008).

Serine-rich repeat adhesins are sialoglycan-binding glycoproteins expressed on the surface of Gram-positive bacteria. They consist of conserved domains including an N-terminal signal peptide, a short SRR region, a ligand-binding basic region (BR) (Jakubovics et al., 2009), a long SRR region, and a C-terminal cell wall-anchoring domain (Bensing et al., 2016). Although the domains are conserved, BRs are highly divergent in amino acid sequence conferring the binding specificity to their cognate ligand (Takamatsu et al., 2005). For instance, Hsa and GspB, which are homologous SRR adhesins expressed on *S. gordonii* CH1 and M99 strains, respectively, have different BR structures with distinct binding ability: Hsa binds to both 3′-sialyllactose and sialyl-T antigen, whereas GspB binds only to sialyl-T antigen (Urano-Tashiro et al., 2016).

Dendritic cells are antigen-presenting cells that link the innate and adaptive immune responses (Steinman, 2006). Under infectious conditions, DCs exert various functions as sentinels; they recognize, phagocytose, and process infecting microbes to present the microbial epitopes to naïve T lymphocytes (Kapsenberg, 2003). Upon sensing antigens, DCs upregulate the expression of MHC proteins and co-stimulatory molecules such as CD40, CD80, and CD86. DCs also produce cytokines such as TNF-α, IL-6, and IL-12p70 that result in activation and differentiation of T lymphocytes. Mature DCs can migrate to draining lymph nodes to present antigens to T lymphocytes and induce antigen-specific adaptive immune responses (Mempel et al., 2004).

Serine-rich repeat adhesins of *S. gordonii* are important for this microbe to bind to host cells. This interaction appears to be critical in bacterial infection and host immunity. In the present study, we investigated the role of *S. gordonii* SRR adhesins, Hsa and GspB, in maturation and activation of human DCs treated with wild-type *S. gordonii* and SRR adhesin-deficient mutant strains.

## Materials and Methods

### Reagents and Chemicals

Ficoll-Paque PLUS was obtained from GE Healthcare (Uppsala, Sweden). Penicillin-streptomycin solution and RPMI-1640 were purchased from Hyclone (Logan, UT, USA). Fetal bovine serum (FBS) was purchased from GIBCO (Grand Island, NY, USA). Recombinant human granulocyte macrophage-colony stimulating factor (GM-CSF) and IL-4 were purchased from R&D Systems (Minneapolis, MN, USA) and CreaGene (Sungnam, Korea), respectively. Anti-human CD14 magnetic particles and anti-human CD3 magnetic particles were purchased from BD Biosciences (San Diego, CA, USA). Dimethyl sulfoxide, Red Blood Cell Lysis Buffer, and cytochalasin D were purchased from Sigma–Aldrich (St. Louis, MO, USA). 5-(and-6)-Carboxyfluorescein diacetate succinimidyl ester (CFDA-SE) was obtained from Molecular Probes (Eugene, OR, USA). PE-labeled anti-human CD83, APC-labeled anti-human CD86, APC-labeled anti-human PD-L1, and APC-labeled anti-human CD25 antibodies were purchased from BioLegend (San Diego, CA, USA). FITC-labeled anti-human HLA-DR, DP, and DQ antibodies for MHC class II were obtained from BD Biosciences. All isotype-matched antibodies were obtained from BioLegend or BD Biosciences. Enzyme-linked immunosorbent assay (ELISA) kits for measuring the concentrations of TNF-α, IL-12p70, and IL-6 were purchased from BioLegend. Todd Hewitt broth was obtained from MB Cell (Seoul, South Korea). Bacto^TM^ yeast extract and Bacto^TM^ agar were purchased from BD Biosciences (Sparks, MD, USA). 3′-Sialyllactose (3′SL) was purchased from Cayman Chemical Company (Ann Arbor, MI, USA).

### Generation of Human Monocyte-Derived DCs

All experiments using human blood were conducted under approval of the Institutional Review Board at Seoul National University, South Korea. The Korean Red Cross provided blood from healthy human donors after obtaining informed consent. Peripheral blood mononuclear cells (PBMCs) were isolated using Ficoll-Paque PLUS, as previously described (Kim et al., 2013). PBMCs were then incubated with CD14 magnetic beads for 30 min at room temperature, followed by separation in a magnetic field to isolate CD14^+^ monocytes. The purified CD14^+^ monocytes were suspended in RPMI-1640 supplemented with 10% FBS, 1% penicillin-streptomycin solution, 5 ng/ml GM-CSF, and 10 ng/ml IL-4 and were seeded in 60-mm cell culture dishes at a density of 2 × 10^6^ cells/ml. The monocytes were cultured for 5 days to differentiate into immature DCs. Culture media supplemented with GM-CSF and IL-4 was changed every 3 days.

### Bacteria and Culture Conditions

Wild-type *S. gordonii* CH1 and M99 strains, the Hsa-deficient mutant strain PS798, and the GspB-deficient mutant strain PS846 were kindly provided by Dr. Paul M. Sullam (University of California at San Francisco). The mutants were generated by double cross-over recombination, as described previously (Bensing et al., 2004; Xiong et al., 2008). All bacteria were cultured in TH media containing 0.5% yeast extract until mid-log phase at 37°C. The mutant strains grew comparably well *in vitro* (data not shown). Bacterial cells were harvested by centrifugation at 8,000 rpm for 10 min at 37°C and were washed with PBS. To prepare stocks of wild-type *S. gordonii* and mutant strains, the bacterial pellet was suspended in 50% glycerol THY media to 5 × 10^8^ CFU/ml and stored at −80°C in a freezer.

### Analysis of Bacterial Adherence and Internalization

To label *S. gordonii* with CFSE, the bacterial pellet was suspended in 1 ml PBS containing 10 μM CFDA-SE and incubated for 15 min at 37°C. The bacterial cells were then washed with PBS. Immature DCs (5 × 10^4^ cells) were incubated with either CFSE-labeled wild-type *S. gordonii* or SRR adhesin-deficient mutant strains at 5 × 10^5^, 5 × 10^6^, or 5 × 10^7^ CFU in 50 μl PBS for 1 h at 4°C or 37°C, respectively. Flow cytometry (FACSCalibur, BD Biosciences) was used to measure bacterial binding at 4°C and internalization at 37°C. All cytometric data were analyzed using FlowJo software (TreeStar, San Carlos, CA, USA).

### Analysis of DC Phenotypes

Immature DCs (2.5 × 10^5^ cells/ml) were stimulated with either wild-type *S. gordonii* or SRR adhesin-deficient mutant (1 × 10^6^ CFU/ml) in the presence of GM-CSF (2.5 ng/ml) and IL-4 (5 ng/ml). After 1 h, gentamycin (200 μg/ml) was added to the culture to prevent the bacterial growth and the DCs were further incubated for 23 h. The DCs were stained with fluorochrome-conjugated monoclonal antibodies specific to CD83, CD86, MHC class II, and PD-L1 for 30 min on ice and washed with PBS. The mean fluorescence intensity (MFI) of DCs was analyzed by FACSCalibur, and all flow cytometry data were analyzed by FlowJo software.

### Quantification of Cytokines

Immature DCs (2.5 × 10^5^ cells/ml) were stimulated with either wild-type *S. gordonii* or an SRR adhesin-deficient mutant (1 × 10^6^ CFU/ml) in the presence of GM-CSF and IL-4. To kill *S. gordonii*, gentamicin was added to the DCs, and cells were further incubated for 23 h. Concentrations of TNF-α, IL-12p70, and IL-6 in the culture supernatants were measured by ELISA kits, as described previously (Lee et al., 2015).

### Co-culture of DCs with Autologous T Lymphocytes

Immature DCs (2.5 × 10^5^ cells/ml) were stimulated with either wild-type *S. gordonii* or an SRR adhesin-deficient mutant (1 × 10^6^ CFU/ml) in the presence of GM-CSF and IL-4. After 1 h, DCs were treated with gentamycin to kill *S. gordonii* and were further incubated for another 15 h. To isolate CD3^+^ T lymphocytes, PBMCs were incubated with anti-human CD3 magnetic particles for 30 min at room temperature. CD3^+^ cells were isolated by positive selection according to the manufacturer’s instruction. To label isolated CD3^+^ T lymphocytes with CFSE, the cells were incubated in RPMI-1640 supplemented with 10% FBS, 1% penicillin-streptomycin solution, and 10 μM CFDA-SE for 15 min at 37°C and then washed with PBS. The CFSE-labeled autologous CD3^+^ T lymphocytes (5 × 10^4^ cells) were mixed with *S. gordonii*-stimulated DCs (5 × 10^4^ cells) for 4 days, and the cells were stained with anti-human CD25 antibody. The proliferative activity and activation marker expression of cells were subsequently analyzed by flow cytometry.

### Statistical Analysis

The statistical difference between experimental groups and the control group was analyzed by Student’s *t*-test. *P*-values less than 0.05 were considered statistically significant. Results are indicated as mean of triplicated measurements ± standard error of the mean (SEM).

## Results

### SRR Adhesin-Deficient *S. gordonii* Exhibits Attenuated Binding and Internalization to DCs Compared to the Wild-Type Strain

**FIGURE 1:**
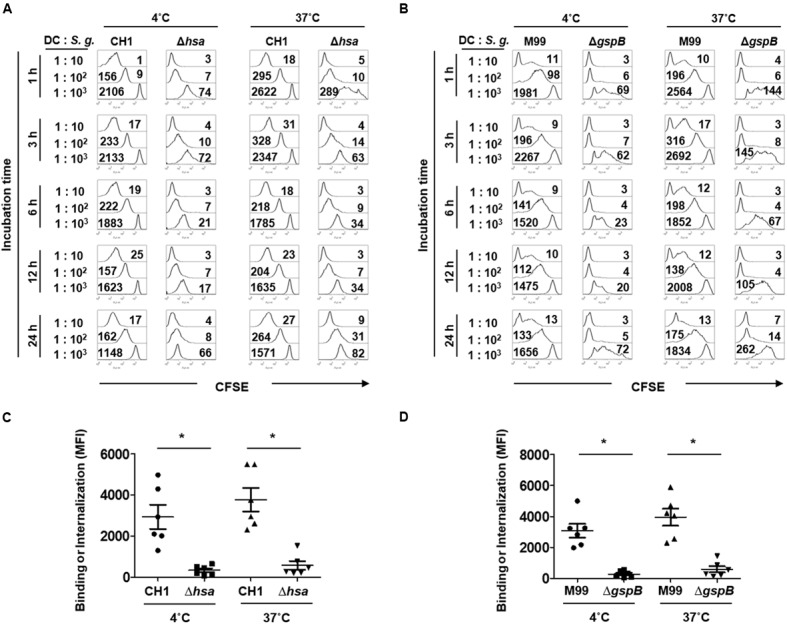
**Serine-rich repeat (SRR) adhesin-deficient *Streptococcus gordonii* exhibits attenuated binding and internalization to dendritic cells (DCs).** Immature DCs (5 × 10^4^ cells) were co-cultured with CFSE-labeled *S. gordonii* CH1 and M99 strains or their SRR adhesin-deficient mutants (Δ*hsa* for CH1 strain and Δ*gspB* for M99 strain) at 1–10, 10^2^, or 10^3^ at 4°C (for binding) and 37°C (for internalization). At 1 h after the treatment, gentamycin (200 μg/ml) was added to prevent the bacterial growth followed by incubation for additional 2, 5, 11, or 23 h. Then, the binding and internalization of **(A)** *S. gordonii* CH1 strain and **(B)** M99 strain to DCs were analyzed by flow cytometry. Immature DCs (5 × 10^4^ cells) were co-cultured with CFSE-labeled *S. gordonii* CH1 and M99 strains or their SRR adhesin-deficient mutants (Δ*hsa* for CH1 strain and Δ*gspB* for M99 strain) at 1–10^2^ at 4°C (for binding) or 37°C (for internalization) for 1 h. The numbers on the histograms indicate the mean fluorescence intensity (MFI). Binding and internalization of **(C)** *S. gordonii* CH1 strain and **(D)** M99 strain to DCs were analyzed by flow cytometry. Graphs of dot plot represent the mean MFI ± SEM from six independent experiments. Asterisk (^∗^) indicates statistical significance (*P* < 0.05).

Bacterial binding and internalization are important processes for DCs to initiate immune responses (Kapsenberg, 2003). The role of SRR adhesins of *S. gordonii* was examined, with focus on Hsa for the CH1 strain and GspB for the M99 strain. Bacterial adherence to DCs was studied at 4°C, while internalization was examined at 37°C. Hsa-deficient *S. gordonii* exhibited attenuated binding and internalizing abilities compared to the wild-type (**Figures 1A,C**). Likewise, GspB-deficient *S. gordonii* more weakly bound and internalized to the DCs than did the wild-type (**Figures 1B,D**). The results indicate that SRR adhesins Hsa and GspB are crucial for the adherence and internalization of *S. gordonii* to DCs.

### SRR Adhesin-Deficient *S. gordonii* More Weakly Increases the Expression of Phenotypic Maturation Markers on DCs than Does the Wild-Type Strain

**FIGURE 2:**
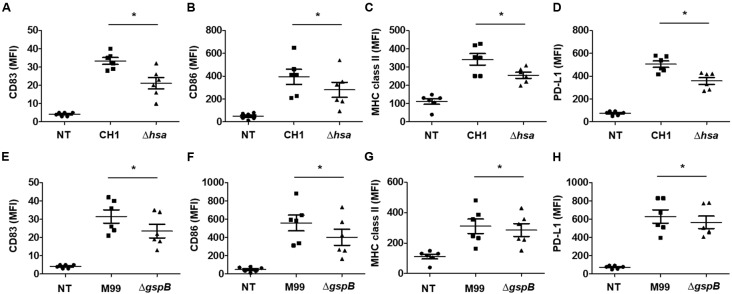
**Serine-rich repeat adhesin-deficient *S. gordonii* weakly induces phenotypic maturation of DCs.** Immature DCs (2.5 × 10^5^ cells/ml) were stimulated with various concentrations of *S. gordonii* CH1 and M99 strains or their SRR adhesin-deficient strains at 1 × 10^6^ CFU/ml for 24 h. Expression of **(A,E)** CD83, **(B,F)** CD86, **(C,G)** MHC class II, and **(D,H)** PD-L1 on DCs was analyzed by flow cytometry. Graphs of dot plot represent the mean MFI ± SEM from six independent experiments. Asterisk (^∗^) indicates statistical significance (*P* < 0.05). NT, non-treatment.

Upon sensing microbial antigens, DCs upregulate a number of molecules such as CD83, CD86, MHC proteins, PD-L1, and PD-L2 to induce an antigen-specific adaptive immune response (Kapsenberg, 2003). To examine the roles of *S. gordonii* Hsa and GspB in inducing phenotypic maturation of DCs, the expression of CD83, CD86, MHC class II, and PD-L1 on DCs upon stimulation with either wild-type *S. gordonii* or an SRR adhesin-deficient mutant was compared. Both wild-type *S. gordonii* CH1 and M99 markedly induced the expression of CD83 (**Figures 2A,E** and Supplementary Figures 1A,E, 2A,E), CD86 (**Figures 2B,F** and Supplementary Figures 1B,F, 2B,F), MHC class II (**Figures 2C,G** and Supplementary Figures 1C,G, 2C,G), and PD-L1 (**Figures 2D,H** and Supplementary Figures 1D,H, 2D,H). However, stimulation with the SRR adhesin-deficient mutant showed lower potency in inducing those molecules (**Figures 2A–H** and Supplementary Figures 1A–H, 2A–H). These results indicate that Hsa and GspB of *S. gordonii* contribute to the expression of activation markers on DCs.

### SRR Adhesin-Deficient *S. gordonii* Induces Cytokine Production Less Potently than the Wild-Type Bacteria

**FIGURE 3:**
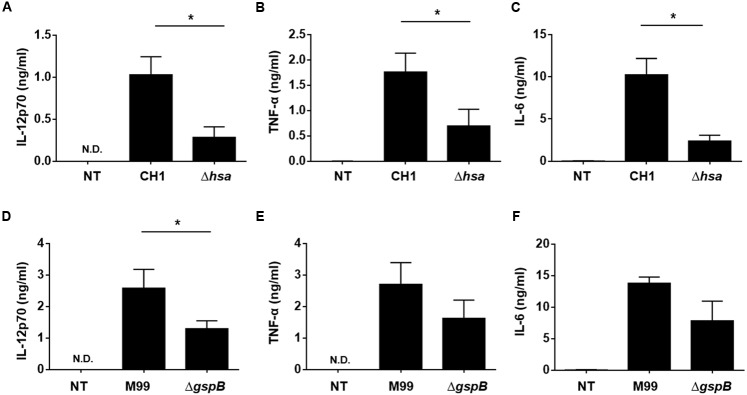
**Serine-rich repeat adhesin-deficient *S. gordonii* more weakly induces DC cytokine production than wild-type *S. gordonii*.** Immature DCs (2.5 × 10^5^ cells/ml) were stimulated with *S. gordonii* CH1 and M99 strains or their SRR adhesin-deficient strains at 1 × 10^6^ CFU/ml for 24 h. The levels of **(A,D)** IL-12p70, **(B,E)** TNF-α, and **(C,F)** IL-6 in the culture media were measured by ELISA. Concentrations of cytokines are indicated as mean value ± SEM from three independent experiments. Statistical difference between experimental groups was analyzed by Student’s *t*-test. *P*-values less than 0.05 were considered statistically significant and are indicated by asterisks (^∗^). N.D., not detected; NT, non-treatment.

When DCs are activated, they express cytokines such as IL-12, IL-10, and TNF-α to mediate inflammatory responses and the activation and differentiation of other immune cells including T lymphocytes (Kapsenberg, 2003). Thus, we next examined cytokine production of DCs induced by stimulation with wild-type *S. gordonii* or SRR adhesin-deficient mutants. DCs stimulated with Hsa-deficient mutant (**Figures 3A–C**) or GspB-deficient mutant (**Figures 3D–F**) resulted in significantly lower production of IL-12p70, TNF-α, and IL-6 in the Hsa-deficient CH1 strain as compared to wild-type. Similarly, a substantially lower secretion of these cytokines was also seen in the GspB-deficient mutant as compared to the M99 WT strain. Neither *S. gordonii* CH1 nor M99 strains induced IL-10 production in DCs (data not shown). These results suggest that SRR adhesins play an important role in *S. gordonii*-induced inflammatory cytokine production by DCs.

### DCs Stimulated with SRR Adhesin-Deficient *S. gordonii* More Weakly Induce Proliferation and Activation of Autologous T Cells than DCs Stimulated with Wild-Type *S. gordonii*

**FIGURE 4:**
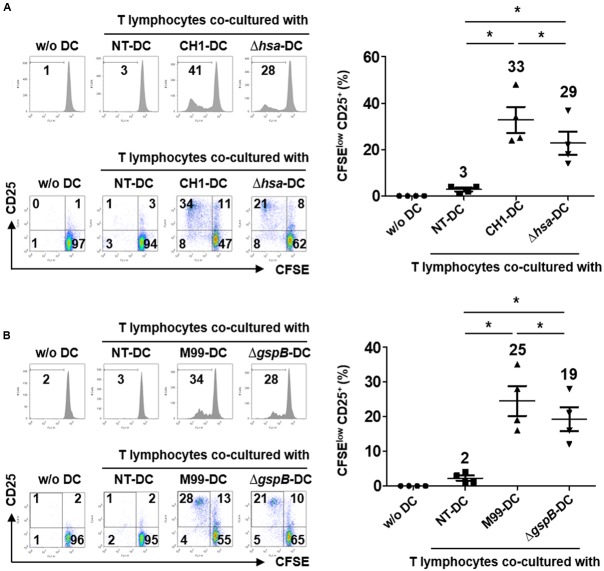
**Dendritic cells stimulated with SRR adhesin-deficient *S. gordonii* more weakly induce proliferation and activation of autologous T cells than DCs stimulated with wild-type *S. gordonii*.** Immature DCs (2.5 × 10^5^ cells/ml) were stimulated with *S. gordonii* CH1 and M99 strains or their SRR adhesin-deficient strains at 1 × 10^6^ CFU/ml for 16 h. DCs were co-cultured with CFSE-labeled autologous T lymphocytes (2.5 × 10^5^ cells/ml) for 5 days. **(A,B)** Proliferation and CD25 expression of T lymphocytes induced by **(A)** DCs sensitized with *S. gordonii* CH1 strain or its mutant and **(B)** DCs sensitized with *S. gordonii* M99 strain or its mutant were examined by flow cytometry. The numbers in each histogram and quadrant indicate the percentage. Histograms represent the proliferation level of T lymphocytes determined by reduced CFSE fluorescence intensity. Graphs of dot plot indicate the mean values of CFSE^low^ CD25^+^ ± SEM from four independent experiments and the actual mean values are on top of each group. Asterisk (^∗^) indicates statistical significance (*P* < 0.05). NT, non-treatment.

Functionally mature DCs exhibit increased expression of MHC proteins, co-stimulatory molecules, and cytokines required for adequate activation of T lymphocytes (Kapsenberg, 2003). To examine the effect of *S. gordonii* SRR adhesins on the T cell-activating capacity of DCs, the proliferative activity and activation marker expression of T lymphocytes were examined through DCs sensitized with wild-type *S. gordonii* or SRR adhesin-deficient mutants. The results showed that Hsa-deficient mutant-sensitized DCs induced proliferation and CD25 expression of T lymphocytes less potently than DCs sensitized with wild-type *S. gordonii* (**Figure 4A**). Likewise, T lymphocytes exhibited weakened proliferative activity and activation marker expression in response to GspB-deficient mutant-sensitized DCs (**Figure 4B**). Taken together, these results indicate that Hsa and GspB contribute to DC-mediated immune activation of T lymphocytes by *S. gordonii*.

### Blockade of SRR Adhesins Abolishes *S. gordonii* Binding and Internalization to DCs and Attenuates Immunostimulating Potency

**FIGURE 5:**
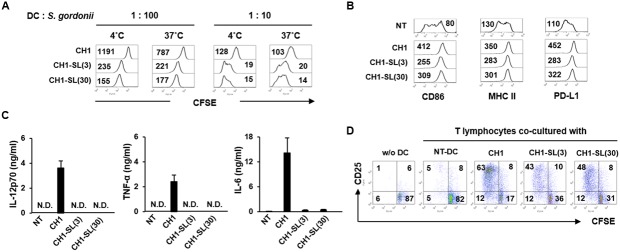
**Blockade of SRR adhesins abolishes *S. gordonii* binding and internalization to DCs and attenuates the immunostimulating potency of bacterium in DCs.** *S. gordonii* CH1 (6.7 × 10^6^ CFU/ml) was pretreated with 3′-sialyllactose (3 and 30 μM) for 1 h at room temperature to block bacterial Hsa and was washed with PBS. **(A)** Immature DCs (5 × 10^4^ cells) were incubated in the absence or presence of 3′-sialyllactose-pretreated *S. gordonii* (5 × 10^5^ and 5 × 10^6^ CFU) in 50 μl PBS for 1 h at 4 and 37°C, respectively. Bacterial binding (4°C) and internalization (37°C) to DCs were analyzed by flow cytometry. The numbers on the histogram indicate the MFI of DCs. **(B,C)** Immature DCs (2.5 × 10^5^ cells/ml) were either untreated or stimulated with 3′-sialyllactose-pretreated *S. gordonii* (1 × 10^6^ CFU/ml) for 24 h. **(B)** Expression of CD86, MHC II, and PD-L1 on DCs was analyzed by flow cytometry. The numbers on the histogram indicate the MFI of DCs. **(C)** Concentrations of IL-12p70, TNF-α, and IL-6 in DC culture supernatants were measured by ELISA. **(D)** *S. gordonii*-sensitized DCs (5 × 10^4^ cells) were co-cultured with autologous CD3^+^ T lymphocytes (5 × 10^4^ cells) for 4 days. Proliferative activity and CD25 expression of T lymphocytes were analyzed by flow cytometry. The numbers in each quadrant indicate the percentage of T lymphocytes. Results are representative of two similar experiments. N.D., not detected; NT, non-treatment; CH1, *S. gordonii* CH1 unpretreated with 3′-sialyllactose; CH1-SL(3), *S. gordonii* CH1 pretreated with 3 μM 3′-sialyllactose; CH1-SL(30), *S. gordonii* CH1 pretreated with 30 μM 3′-sialyllactose.

Hsa, the SRR adhesion of *S. gordonii* CH1 strain, specifically bind to 3′SL (Urano-Tashiro et al., 2016). In order to further examine the role of Hsa in the bacterial interaction with DCs, *S. gordonii* CH1 strain pretreated with 3′SL (named CH1-SL) was treated with DCs followed by analysis of the bacterial adherence to DCs and phenotypic and functional maturation of DCs. CH1-SL showed marked attenuation in binding and internalizing DCs (**Figure 5A**). In addition, pretreatment with 3′SL abolished the immunostimulatory potency of *S. gordonii*. DCs stimulated with CH1-SL exhibited lower expression of maturation markers such as CD86, MHC class II, and PD-L1 than DCs stimulated with unpretreated *S. gordonii* (**Figure 5B**). Furthermore, stimulation with CH1-SL diminished the production of IL-12p70, TNF-α, and IL-6 by DCs (**Figure 5C**). When CH1-SL-sensitized DCs were co-cultured with autologous T lymphocytes, proliferation and activation of T lymphocytes were more weakly induced than with DCs sensitized by unpretreated *S. gordonii* (**Figure 5D**). These results suggest that Hsa is a primary target molecule mediating *S. gordonii* binding and internalization to DCs and the immunostimulatory potency of the bacteria in DC maturation.

## Discussion

*Streptococcus gordonii* SRR adhesins, Hsa and GspB, are important not only for bacterial adhesion to host cells, but also for activation of host immune responses. Here, we demonstrated that *S. gordonii* lacking SRR adhesins showed marked reduction in DC maturation, production of inflammatory cytokines, and T cell-activating ability compared to wild-type *S. gordonii*. These results suggest that SRR adhesins Hsa and GspB are major surface molecules that are responsible for *S. gordonii*-induced DC maturation and activation.

Intact *S. gordonii* appears to induce the maturation and activation of human DCs, which is coincident with previous findings that phenotypic maturation and cytokine production take place in murine and human DCs stimulated with *S. gordonii* (Corinti et al., 1999; Mayer et al., 2009). On the other hand, IL-10 was hardly induced in human DCs treated with *S. gordonii* while other cytokines such as TNF-α, IL-6, and IL-12 were substantially increased under the same condition. Interestingly, however, previous reports demonstrated IL-10 induction in human DCs treated with *S. gordonii* (Corinti et al., 1999, 2001). Some possible explanations for this discrepancy can be made. One might be the difference in the intrinsic property of DCs originated from blood of humans with different ethnic backgrounds. Another might be the difference in the experimental conditions used for the preparation of DCs such as the separation method of DC precursors, the concentration of GM-CSF and IL-4, and the culture media composition containing supplementary ingredients. The third one might be the difference in the bacteria-to-DC ratio. The previous report demonstrated no induction of IL-10 at *S. gordonii*-to-DC ratio of 1:1 while a small amount of IL-10 (<100 pg/ml) at 10:1 (Corinti et al., 1999). Another study showed that *S. gordonii*-to-DC ratio of 50:1 was comparable to *S. typhi*-to-DC ratio of 1:1 for the induction of IL-10 at the similar extent, implying the low potentcy of *S. gordonii* to induce IL-10 in DCs (Corinti et al., 2001). Therefore, our results showing no IL-10 induction could be due to the difference in the intrinsic property of DCs derived from different ethnic background, the experimental method, and/or the use of low *S. gordonii*-to-DC ratio at 4:1.

We used SRR adhesin-deficient mutant strains to demonstrate that SRR adhesins Hsa and GspB contribute to *S. gordonii*-induced DC maturation and activation. This is in line with previous findings that *S. gordonii* binding to human monocytes via Hsa promoted their differentiation into DCs (Urano-Tashiro et al., 2012). Interestingly, other surface adhesins of *S. gordonii* are also involved in the activation of innate immune cells. SspA and SspB, famous adhesins of *S. gordonii*, are the best examples for the induction of cytokines in epithelial cells and DCs (Andrian et al., 2012). We suggest that the SRR adhesins of *S. gordonii* are not only involved in bacterial adherence, but also actively contribute to the induction of innate immunity by maturation and activation of DCs.

Although SRR-deficient *S. gordonii* showed a decreased augmentation on the expression of maturation markers including CD83, CD86, MHC class II, and PD-L1, in comparison with the wild-type strain, the difference between the wild-type and mutant appears to vary in each marker. It is likely due to the difference in the signaling pathways coincident with differential induction and turn-over rate. Furthermore, the more bacteria were treated, the less difference was observed in their expression. It may be because *S. gordonii* also possesses other immunostimulatory molecules such as lipoprotein and LTA in the cell wall that are known to involve the expression of the maturation markers on the host immune cells (Chan et al., 2007; Cho et al., 2013). On the other hand, it is notable that the loss of Hsa in *S. gordonii* CH1 strain was more dramatic than the loss of GspB in *S. gordonii* M99 strain in the phenotypic and functional maturation of DCs. The differential profiles of DC maturation might be due to the difference in the ligand-binding BR structure and glycan specificity between GspB and Hsa (Bensing et al., 2016) as demonstrated by the previous study that Hsa binds to both 3′SL and sialyl-T antigen, whereas GspB binds only to sialyl-T antigen (Urano-Tashiro et al., 2016).

Accumulating reports suggest that *S. gordonii* exhibit similar properties with regard to binding and internalization in various cell types, including monocytes, macrophages, erythrocytes, and platelets (Kerrigan et al., 2007; Urano-Tashiro et al., 2008). For example, *S. gordonii* binds to membrane glycoprotein Ibα on human platelets through bacterial Hsa or GspB, and the lack of GspB decreased platelet binding of the *S. gordonii* M99 strain by approximately 70% (Takamatsu et al., 2005; Xiong et al., 2008). Concordant with previous reports, the current results also showed that *S. gordonii* lacking Hsa or GspB was not efficiently adhered or internalized to DCs, leading to insufficient maturation and activation of DCs. These results support the hypothesis that SRR adhesins Hsa and GspB are important for the interaction of *S. gordonii* and DCs, which stimulate innate immunity mediated through DCs.

Serine-rich repeat adhesins were reported to bind to sialic acids of host cells, contributing to the pathogenesis of *S. gordonii* (Urano-Tashiro et al., 2008). Indeed, *S. gordonii* CH1 exhibited markedly attenuated binding and internalizing ability to DCs in the presence of 3′SL, which might interfere with the interaction of the bacterial Hsa with sialylated motifs on DCs. Moreover, the bacteria pretreated with 3′SL showed weakened induction of maturation, cytokine production, and T cell-activating ability of DCs. Interestingly, the inhibitory effect of 3′SL was dramatic on the induction of cytokine production in comparison with that of phenotypic marker expression. Those differential effects could be due to the distinct intracellular signal transduction pathways required for the expression of cytokines and co-stimulatory receptors. For example, the expression of co-stimulatory receptors including CD80 and CD86 was highly induced by lipopolysaccharide without induction of TNF-α or IL-12 in MyD88-deficient DCs (Kaisho et al., 2001). Therefore, we speculate that the bacterial interaction through SRR with DCs could predominantly participate in the stimulation of signaling pathways for the induction of cytokines rather than phenotypic markers in DCs.

Bacterial binding and internalization are important steps for DC maturation. Many previous studies have reported that a blockade of bacterial adherence and internalization to DCs attenuated the phenotypic and functional activation of DCs. One study showed that clinically isolated Group A Streptococcus did not induce maturation of DCs when binding and/or internalization was perturbed by bacterial hyaluronic acid capsular polysaccharides (Cortes and Wessels, 2009). In addition, encapsulated *Klebsiella pneumoniae* hardly induced DC maturation because a thick capsule layer on the bacterial surface hindered its phagocytosis by Evrard et al. (2010). Consistent with these reports, our findings showed that SRR adhesin-deficient *S. gordonii* mutant strains had weak binding and internalizing abilities. This consequently induced phenotypic and functional activation of DCs to a lesser extent. Furthermore, the inhibition of bacterial internalization with cytochalasin D abrogated *S. gordonii*-induced maturation and activation of DCs (data not shown). Therefore, Hsa- or GspB-mediated binding and internalization of *S. gordonii* to DCs could be the important step for DC maturation and activation.

Although oral streptococci are considered normal flora of the human oral cavity, some have recently been suggested as etiologic agents for systemic diseases including infective endocarditis and osteomyelitis (Li et al., 2000). It is important to understand the exact pathogenic mechanisms of oral bacteria and to characterize their major virulence factors in order to develop preventive and therapeutic agents against *S. gordonii* infection. Because DCs are the primary sentinel cells used to monitor infections and bridge innate and adaptive immunity for host protection, the SRR adhesins of *S. gordonii* might be major immunomodulatory molecules. Further studies are needed to identify the DC receptors that specifically bind to Hsa and GspB and elucidate downstream signal pathways to activate DCs. The results suggest that the SRR adhesins of *S. gordonii* are major virulence factors involved in bacterial adherence to the host and also trigger DC maturation and activation.

## Author Contributions

SH conceived the idea and contributed to the discussion of the results followed by writing and reviewing the manuscript. SH, EBK, and SK designed the experiments, performed the experiments, and/or interpreted the data. HS and C-HY provided critical comments and contributed to the discussion of the results followed by writing and reviewing the manuscript.

## Conflict of Interest Statement

The authors declare that the research was conducted in the absence of any commercial or financial relationships that could be construed as a potential conflict of interest.
