# Supplementary material for: Serine-Rich Repeat Adhesins Contribute to Streptococcus gordonii-Induced Maturation of Human Dendritic Cells
Source: Front Microbiol. 2017 Mar 31;8:523. doi: 10.3389/fmicb.2017.00523 (PMC5374164; doi:10.3389/fmicb.2017.00523)
Supplement: Supplementary file 1 [file Data_Sheet_1.DOCX]

Supplementary Material

Serine-Rich Repeat Adhesins Contribute to *Streptococcus gordonii*-induced Maturation of Human Dendritic Cells

**Eun Byeol Ko, Sun Kyung Kim, Ho Seong Seo, Cheol-Heui Yun, Seung Hyun Han^*^**

*** Correspondence:** Seung Hyun Han: [shhan-mi@snu.ac.kr](mailto:shhan-mi@snu.ac.kr)

**Materials and Methods**

**Generation of Human Monocyte-Derived DCs**

All experiments using human blood were conducted under approval of the Institutional Review Board at Seoul National University, Republic of Korea. The Korean Red Cross provided blood from healthy human donors after obtaining informed consent. Peripheral blood mononuclear cells (PBMCs) were isolated using Ficoll-Paque PLUS, as previously described (Kim et al., 2013). PBMCs were then incubated with CD14 magnetic beads for 30 min at room temperature, followed by separation in a magnetic field to isolate CD14^+^ monocytes. The purified CD14^+^ monocytes were suspended in RPMI-1640 supplemented with 10% FBS, 1% penicillin-streptomycin solution, 5 ng/ml GM-CSF, and 10 ng/ml IL-4 and were seeded in 60-mm cell culture dishes at a density of 2 × 10^6^ cells/ml. The monocytes were cultured for 5 days to differentiate into immature DCs. Culture media supplemented with GM-CSF and IL-4 was changed every 3 days.

**Bacteria and Culture Conditions**

Wild-type *S. gordonii* CH1 and M99 strains, the Hsa-deficient mutant strain PS798, and the GspB-deficient mutant strain PS846 were kindly provided by Dr. Paul M. Sullam (University of California at San Francisco). The mutants were generated by double cross-over recombination, as described previously (Bensing et al., 2004; Xiong et al., 2008). All bacteria were cultured in TH media containing 0.5% yeast extract until mid-log phase at 37˚C. The mutant strains grew comparably well *in vitro* (data not shown). Bacterial cells were harvested by centrifugation at 8,000 rpm for 10 min at 37˚C and were washed with PBS. To prepare stocks of wild-type *S. gordonii* and mutant strains, the bacterial pellet was suspended in 50% glycerol THY media to 5 × 10^8^ CFU/ml and stored at -80˚C in a freezer.

**Analysis of DC Phenotypes**

Immature DCs (2.5 × 10^5^ cells/ml) were stimulated with either wild-type *S. gordonii* or SRR adhesin-deficient mutant (1 × 10^6^ CFU/ml) in the presence of GM-CSF (2.5 ng/ml) and IL-4 (5 ng/ml). After 1 h, gentamycin (200 µg/ml) was added to the culture to prevent the bacterial growth and the DCs were further incubated for 23 h. The DCs were stained with fluorochrome-conjugated monoclonal antibodies specific to CD83, CD86, MHC class II, and PD-L1 for 30 min on ice and washed with PBS. The mean fluorescence intensity (MFI) of DCs was analyzed by FACSCalibur, and all flow cytometry data were analyzed by FlowJo software.


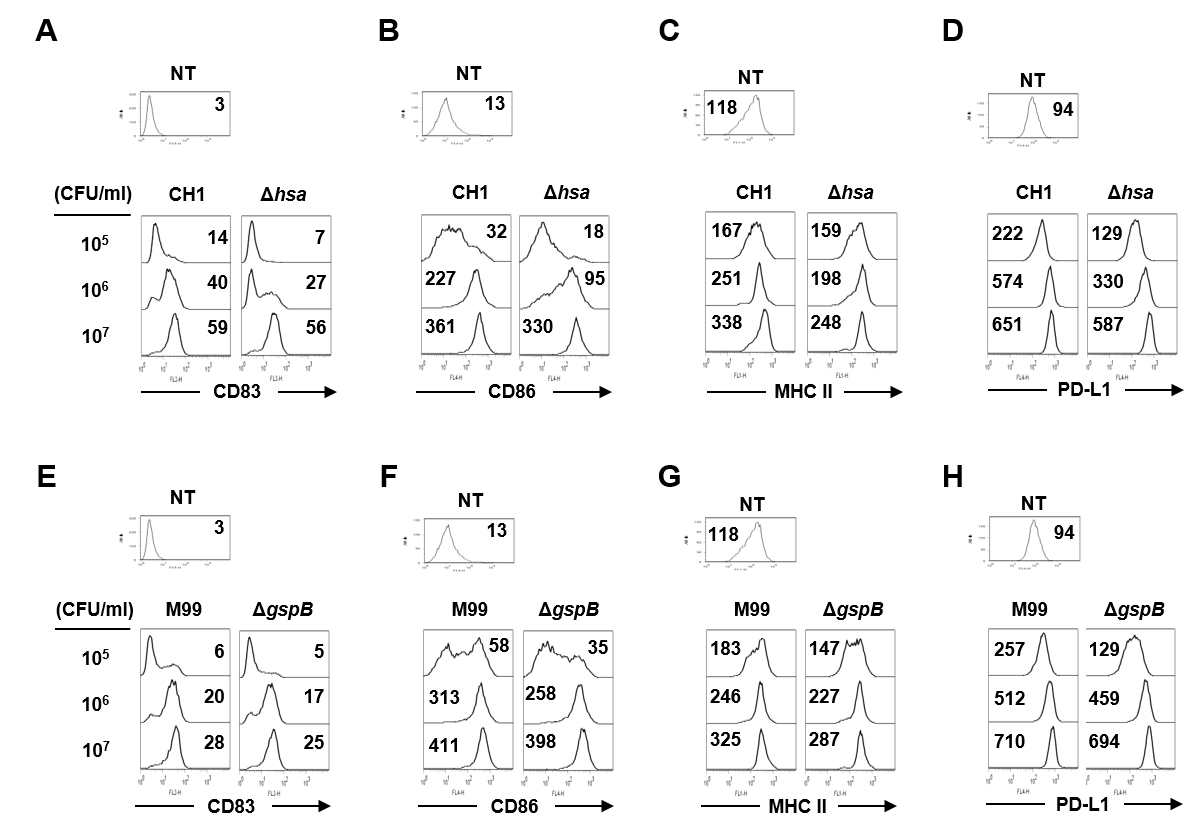


**Supplementary Figure 1.** **SRR adhesin-deficient *S. gordonii* weakly induces phenotypic maturation of DCs in a dose-dependent manner.**

Immature DCs (2.5 × 10^5^ cells/ml) were stimulated with various concentrations of *S. gordonii* wild-type or SRR adhesin-deficient strains (1 × 10^5^, 1 × 10^6^, or 1 × 10^7^ CFU/ml). After 1 h, gentamycin (200 µg/ml) was added to the culture to prevent the bacterial growth and the DCs were further incubated for 23 h. Expression of (A, E) CD83, (B, F) CD86, (C, G) MHC class II, and (D, H) PD-L1 on DCs stimulated with *S. gordonii* CH1 strain or M99 strain was analyzed by flow cytometry. NT, non-treatment.


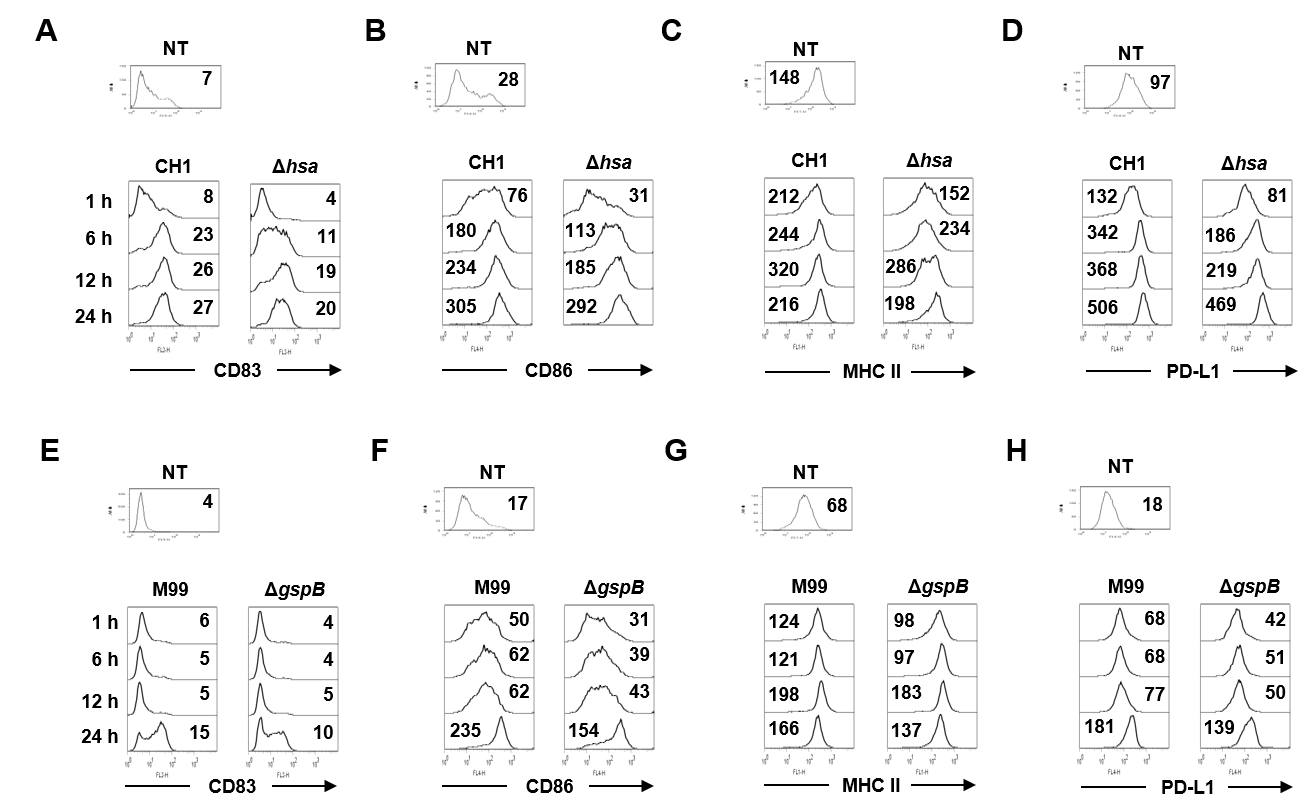


**Supplementary Figure 2.** **SRR adhesin-deficient S. gordonii weakly induces phenotypic maturation of DCs in a time-dependent manner.**

Immature DCs (2.5 × 10^5^ cells/ml) were stimulated with *S. gordonii* CH1 and M99 strains or their SRR adhesin-deficient strains (1 × 10^6^ CFU/ml). After 1 h, gentamycin (200 µg/ml) was added to the culture to prevent the bacterial growth and the DCs were further incubated for additional 0, 5, 11, or 23 h. Expression of (A, E) CD83, (B, F) CD86, (C, G) MHC class II, and (D, H) PD-L1 on DCs stimulated with *S. gordonii* CH1 strain or M99 strain was analyzed by flow cytometry. The numbers on the histograms indicate MFIs. Results are representative of seven similar independent experiments. NT, non-treatment.
